# New western Palaearctic *Dinotrema* species with mesoscutal pit and only medially sculptured propodeum (Hymenoptera, Braconidae, Alysiinae)

**DOI:** 10.3897/zookeys.260.4084

**Published:** 2013-01-21

**Authors:** Thorkild Munk, Francisco Javier Peris-Felipo, Ricardo Jiménez-Peydró

**Affiliations:** 1 Natural History Museum Århus, Denmark; 2 Laboratory of Entomology and Pest Control, Institute Cavanilles of Biodiversity and Evolutional Biology, University of Valencia, c/.Catedrático José Beltrán n°2, 46980 Paterna, Valencia, Spain

**Keywords:** Braconidae, Alysiinae, *Dinotrema*, new species, western Palaearctic

## Abstract

Descriptions of four new species of the genus *Dinotrema* Foerster with a mesoscutal pit and only medially sculptured propodeum are given. *Dinotrema alysiae* **sp. n. (**Denmark, England, Netherlands, Spain), *Dinotrema paramicum* **sp. n.** (Denmark, Finland), *Dinotrema tirolense* **sp. n.** (Italy) and *Dinotrema valvulatum* **sp. n.** (Denmark, Italy).

## Introduction

The genus *Dinotrema* Foerster, 1862 is the largest genus of the subfamily Alysiinae with approximately 320 species described worldwide (Yu et al. 2005). About 250 species of this genus were recorded in the European fauna (Fischer 1972, 1973a, 1993, 1996; van Achterberg 1988; Tobias 2003, 2004a, 2004b, 2006, etc). In spite of this number, numerous Palaearctic *Dinotrema* species remain undescribed up to now.

The current status of the genus *Dinotrema* was established by van Achterberg (1988), and this genus differs from the closely related *Aspilota* Foerster, 1862 in the size of paraclypeal areas which are not connected to the inner eye margin. *Dinotrema* species are parasitoids of Diptera mainly belonging to the family Phoridae (van Achterberg 1988) as well as Anthomyiidae and Platypezidae (Fischer et al. 2008).

We have revised the available type material of this genus of the European fauna to estimate the real composition of *Dinotrema* species and prepare a new determination key for these species. In this paper, four new species with a mesoscutal pit and only medially sculptured propodeum are described and illustrated, viz. *Dinotrema alysiae* sp. n., *Dinotrema paramicum* sp. n., *Dinotrema tirolense* sp. n., and *Dinotrema valvulatum* sp. n.

For the terminology of the morphological features and sculpture, measurements and wing venation nomenclature, see Fischer (1973b). The following abbreviations, generally accepted in the taxonomy of Hymenoptera, are used in the paper: POL – postocellar line; OOL – ocular-ocellar line; OD – maximum diameter of lateral ocellus. The types of species described are deposited in the following museums: Entomological Collection of the University of Valencia (Valencia, Spain; further – ENV), Natural History Museum (London, England; – BMNH), Naturhistorisk Museum (Århus, Denmark; further – NMA), Naturalis Biodiversity Center (Leiden, Netherlands; further – RMNH) and Zoologische Staatssammlung München (Germany; further – ZSSM).

## Taxonomical part

### Dinotrema alysiae

Munk & Peris-Felipo sp. n.

urn:lsid:zoobank.org:act:144B8729-5997-4CF8-819C-AD584000E657

http://species-id.net/wiki/Dinotrema_alysiae

Figs 1 [Fig F2] –13

#### Type material.

Holotype, female (NMA), “Denmark, E-Jutland, Mols Strandkær, 56°14'N, 10°25'E, 02.09.1991, Munk”. Paratypes: 2 females (NMA), “same label as holotype but, 30.07.1991, Munk”; 2 females (NMA), “Denmark, E-Jylland, Yoling Skov sw. of Skanderborg, 06.09.1986, Munk”; 1 female (RMNH), Netherlands, Waarder (Z.H.), Oosteinde, 30–31.08.1974, C. v. Achterberg”; 2 females (BMNH), “England, Bramham Park Nat., Hants., ex. *Callomyia amoena*, 1985, R.E. Evans”.

#### Other material:

1 female (ENV), “Denmark, E-Jutland, Højkol Skov, 56°05'N, 9°38'E, 11.09.2000, Munk”; 1 female (ENV), “Spain, Navarra, Artikutza, Mixto M-1, 16.10.1995, L. Murguia”; 1 female (ENV), “Spain, Navarra, Artikutza, Mixto M-2, 24.07.1995, L. Murguia”.

#### Diagnosis.

This new species resembles *Dinotrema erythropum* Foerster and *Dinotrema valvulatum* sp. n. *Dinotrema alysiae* sp. n. differs from *Dinotrema erythopum* in having the first flagellar segments 3.50 times as long as wide (2.50 times in *Dinotrema erythopum*), middle flagellar segments 1.90–2.00 times as long as wide (1.40 times in *Dinotrema erythropum*), first metasomal tergite 1.45 times as long as apical width (1.70 times in *Dinotrema erythropum*), mesoscutal pit oval (slender and very long in *Dinotrema erythropum*), and lower tooth shorter than upper tooth (longer in *Dinotrema erythropum*). The new species differs from *Dinotrema valvulatum* in having the first metasomal tergite almost entirely smooth (sculptured with two dorsal carinae in *Dinotrema valvulatum*) and ovipositor distinctly shorter than metasoma (ovipositor as long as metasoma in *Dinotrema valvulatum*).

#### Description.

Holotype, female, length of body 2.30–2.40 mm, of fore wing 3.30–3.35 mm.

*Head*. In dorsal view, 1.85–1.90 times as wide as its median length, 1.40 times as wide as mesoscutum, smooth, with rounded temples behind eye. Eye in lateral view 1.60–1.65 times as high as wide and 1.05–1.10 times as wide as temple. POL 3.15–3.20 times OD; OOL 3.25–3.30 times OD. Face 1.80–2.00 times as wide as high; inner margins of eyes subparallel. Clypeus 1.90–1.95 times as wide as high, slightly curved ventrally. Diameter of paraclypeal fovea half distance between clypeus and eye. Mandible widened towards apex, 1.50 times as long as its maximum width. Upper tooth weakly shorter and as wide as base of middle tooth. Middle tooth the longest, wide basally and pointed apically. Lower tooth rather long, but weakly shorter and wider than upper tooth, rounded apically. Antenna thick, 24–25-segmented. Scape 2.40–2.45 times as long as pedicel. First flagellar segment 3.50 times as long as its apical width, 1.10–1.15 times as long as second segment; second segment 2.60–2.65 times as long as its maximum width. Third to twenty-second flagellar segments 1.90–2.00 times as long as their width; twenty-third segment 2.40 times as long as wide.

*Mesosoma*. In lateral view, 1.30 times as long as high. Mesoscutum 1.05–1.10 times as long as maximum width, with two rows of two setae. Notauli mainly absent. Mesoscutal pit present, oval. Prescutellar depression smooth, with lateral carinae. Sternaulus (= precoxal suture) present, not reaching anterior and posterior parts of mesopleuron. Posterior mesopleural furrow smooth below. Propodeum with median longitudinal carina running from anterior to posterior margin, in anterior third with transverse angulated carina, with additional long subparallel carinae laterally to median one; from lateral carinae emerging short carinae not reaching with the propodeal edges. Propodeal spiracles relatively small.

*Legs*. Hind femur 4.10 times as long as wide. Hind tibia weakly widened to apex, 9.10 times as long as its maximum subapical width, 1.10 times as long as hind tarsus. First segment of hind tarsus 2.65 times as long as second segment.

*Wings*. Length of fore wing 2.35–2.40 times its maximum width. Vein r1 present. Radial cell reaching to apex of wing, 3.40–3.45 times as long as its maximum width. Nervulus distinctly postfurcal. Brachial cell closed, 3.25 times as long as its maximum width. Hind wing 5.00 times as long as its maximum width.

*Metasoma*. Distinctly compressed. First tergite weakly widened towards apex, 1.45 times as long as its apical width, almost entirely smooth. Ovipositor as long as first tergite, shorter than metasoma, 0.40–0.45 times as long as hind femur.

*Colour*. Body, mandible and first metasomal tergite dark brown. Legs brown. Wings hyaline. Pterostigma brown.

Male unknown.

**Etymology.** The name is referring to the general size and shape of the body which is very similar as species of *Alysia* genus.

**Figure 1.**
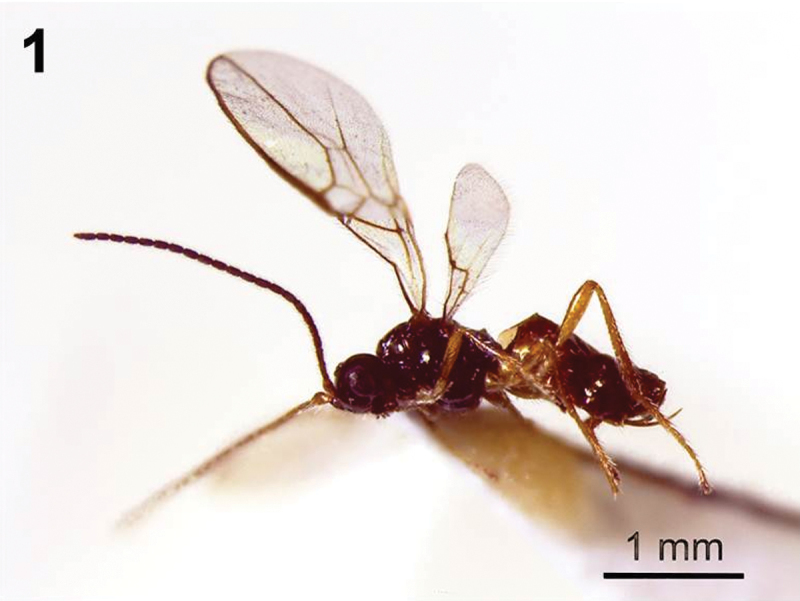
*Dinotrema alysiae* sp. n. (female).Habitus, lateral view.

**Figures 2–7. F2:**
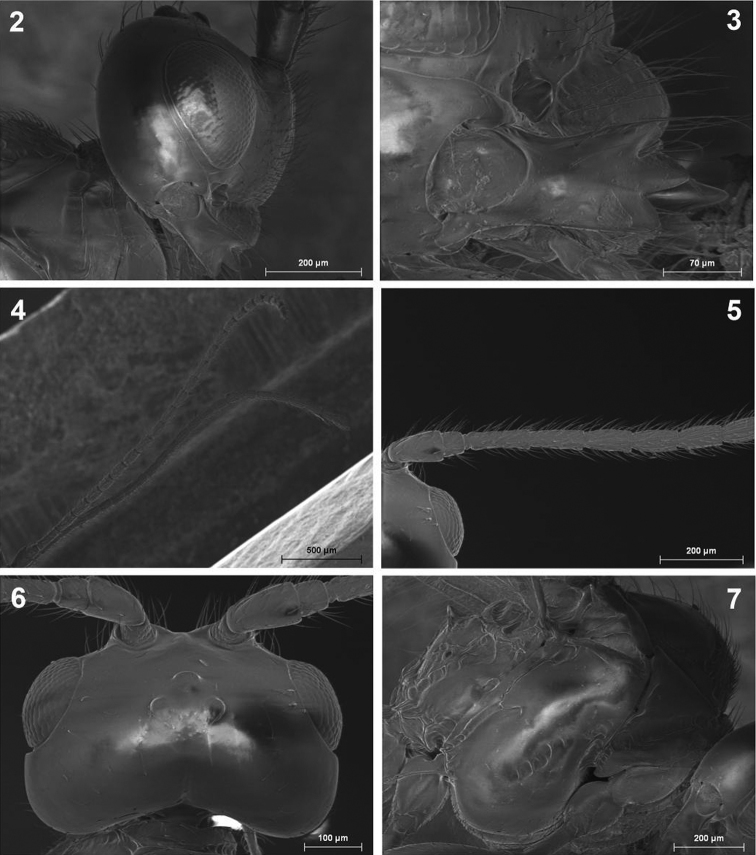
*Dinotrema alysiae* sp. n. (female). **2** Head, lateral view **3** Mandible **4** Antenna **5** Basal segments of antenna **6** Head, dorsal view **7** Mesosoma.

**Figures 8–13.**
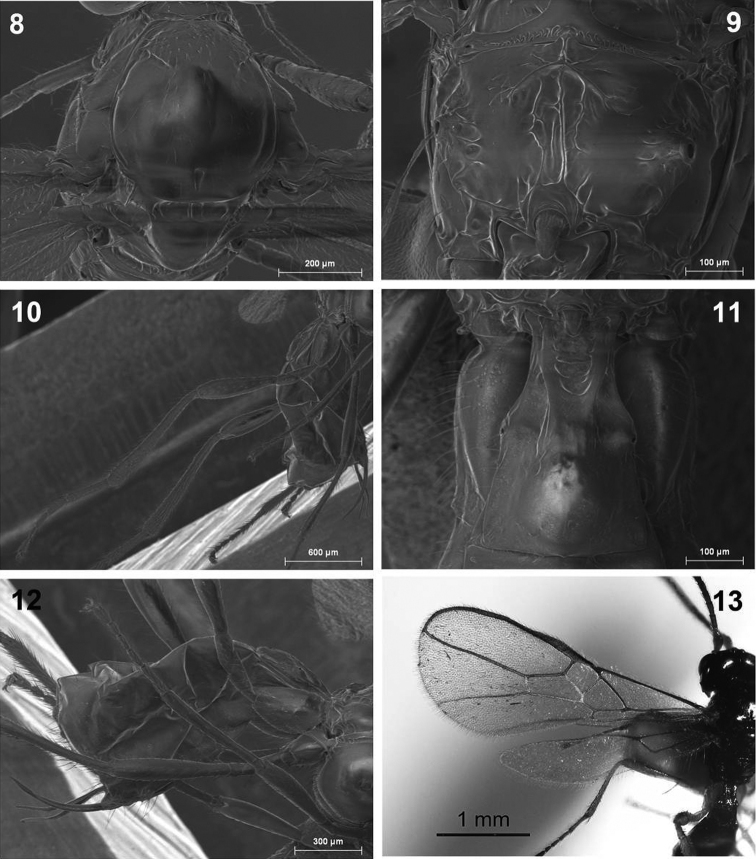
*Dinotrema alysiae* sp. n. (female). **8** Mesonotum **9** Propodeum **10** Hind leg **11** First metasomal tergite **12** Metasoma and ovipositor **13** Fore and hind wings.

### Dinotrema paramicum

Munk & Peris-Felipo sp. n.

urn:lsid:zoobank.org:act:CF5FE99B-66C1-43FF-8E16-1AE7409577E5

http://species-id.net/wiki/Dinotrema_paramicum

**Figure 14.**
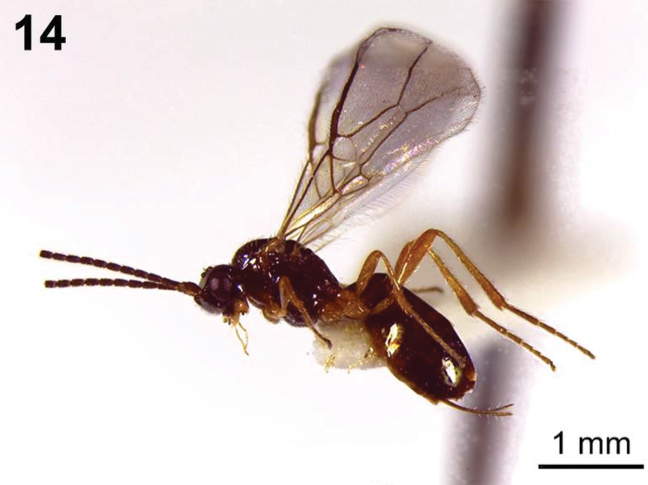
*Dinotrema paramicum* sp. n. (female). **14** Habitus, lateral view

Figs 14 [Fig F5] –26

#### Type material.

Holotype: 1 female (NMA), “Denmark, E-Jutland, Mols, Strandkær 56°14'N, 10°25'E, 30.09.1982, Munk”. Paratypes: 2 females (NMA), same label as holotype; 1 female (ENV), same label as holotype; 1 female (NMA), “Finland, E.S. Ristlina, 6826:501, 06.08.1978, M. Koponen”; 1 female (ENV), “Finland, U. Nurmijärvi, 6715:376, 05.08.1976, M. Koponen”.

#### Diagnosis.

This new species resembles *Dinotrema kempei* (Hedqvist) but differs in having first metasomal tergite 2.30–2.35 times as long as its apical width (3.50-4.00 times in *Dinotrema kempei*), mesoscutal pit rounded (elongated in *Dinotrema kempei*), prescutellar depression rectangular and without lateral carinae (square and with lateral carinae in *Dinotrema kempei*) and mesoscutum with two rows of double setae (with two rows of single setae in *Dinotrema kempei*).

#### Description.

Holotype, female, length of body 2.70–2.80 mm, of fore wing 3.25–3.30 mm.

*Head*. In dorsal view, 1.60–1.65 times as wide as its median length, 1.45–1.50 times as wide as mesoscutum, smooth, with rounded temples behind eye. Eye in lateral view 1.75 times as high as wide and 0.90–0.95 times as wide as temple. POL 2.60–2.65 times OD; OOL 2.60–2.65 times OD. Face 1.60 times as wide as high; inner margins of eyes subparallel. Clypeus 2.65 times as wide as high, slightly curved ventrally. Diameter of paraclypeal fovea less than half of distance between clypeus and eye. Mandible widened towards apex, 1.20 times as long as its maximum width. Upper tooth distinctly shorter and wider than middle tooth and wider than lower tooth. Middle tooth the longest, wide basally and pointed apically. Lower tooth rounded apically and longer than upper tooth. Antenna thick, 23-segmented, as long as body. Scape 1.55–1.60 times as long as pedicel. First flagellar segment 3.00 times as long as its apical width, 1.05–1.10 times as long as second segment; second segment 2.35 times as long as its maximum width. Third to twentieth flagellar segments 1.70–1.80 times as long as their width; twenty-first segment 2.20 times as long as wide.

*Mesosoma*. In lateral view, 1.10–1.15 times as long as high. Mesoscutum 1.10 times as long as maximum width with two rows of double setae. Notauli mainly absent. Mesoscutal pit present, rounded. Prescutellar depression smooth, without lateral carinae. Sternaulus (= precoxal suture) present, not reaching anterior and posterior parts of mesopleuron. Posterior mesopleural furrow smooth. Propodeum with median longitudinal carina running from anterior to posterior margin. Propodeal spiracles small.

*Legs*. Hind femur 4.15–4.20 times as long as wide. Hind tibia weakly widened to apex, 9.75 times as long as its maximum subapical width, 1.05–1.10 times as long as hind tarsus. First segment of hind tarsus 1.85 times as long as second segment.

*Wings*. Length of fore wing 2.60–2.70 times its maximum width. Vein r1 present. Radial cell reaching to apex of wing, 3.95–3.40 times as long as its maximum width. Nervulus distinctly postfurcal. Brachial cell closed, widened apically, 3.40 times as long as its maximum width. Hind wing 4.50–4.60 times as long as its maximum width.

*Metasoma*. Distinctly compressed. First tergite weakly widened towards apex, 2.80 times as long as its apical width, almost entirely smooth. Ovipositor 1.90–1.95 times as long as first tergite, shorter than metasoma, 1.55–1.60 times as long as hind femur.

*Colour*. Body and legs brown to dark brown. Wings hyaline. Pterostigma brown.

Male unknown.

#### Etymology.

The name is from Greek “para” meaning “elongate” and “micus” from Latin meaning “character” and referring to the general shape of the body.

**Figures 15–20. F5:**
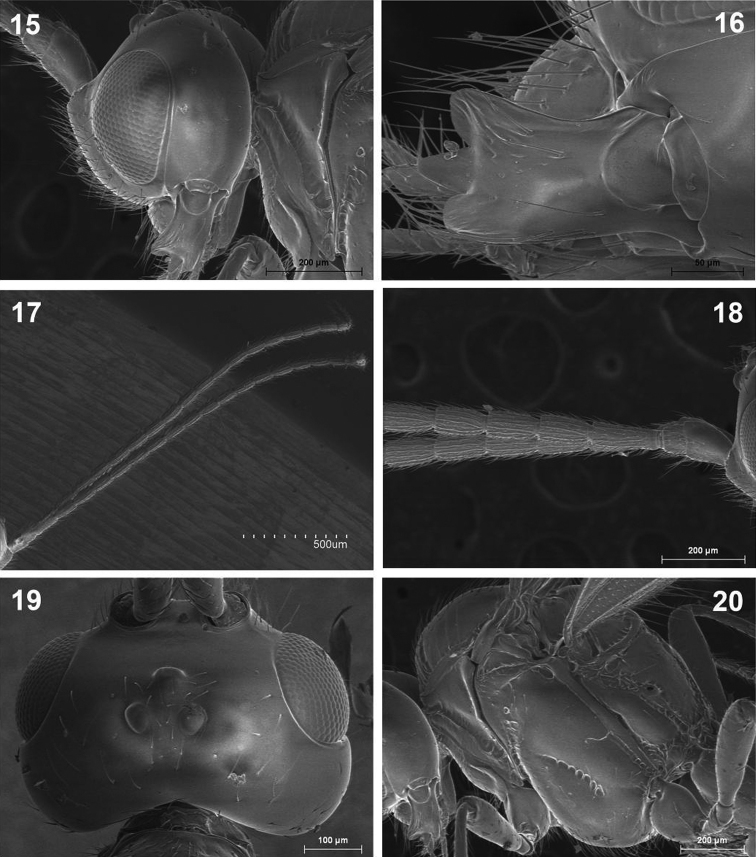
*Dinotrema paramicum* sp. n. (female). **15** Head, lateral view **16** Mandible **17** Antenna **18** Basal segments of antenna **19** Head, dorsal view **20** Mesosoma.

**Figures 21–26.**
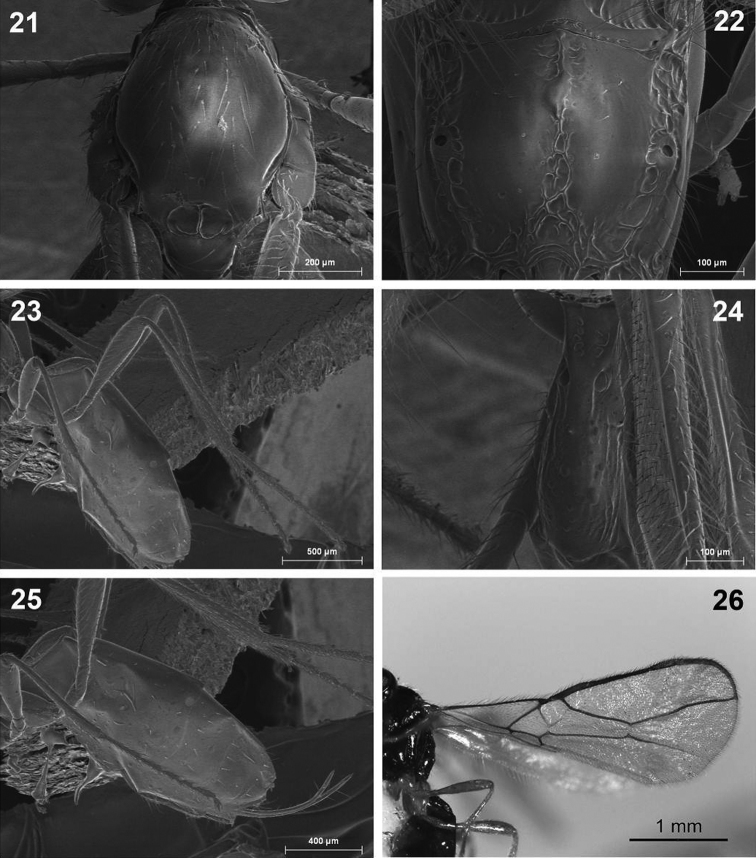
*Dinotrema paramicum* sp. n. (female). **21** Mesonotum **22** Propodeum **23** Hind leg **24 **First metasomal tergite **25** Metasoma and ovipositor **26** Fore and hind wings.

### Dinotrema tirolense

Munk & Peris-Felipo sp. n.

urn:lsid:zoobank.org:act:13DD07E4-4DDA-469E-9FE9-428DA8CD0504

http://species-id.net/wiki/Dinotrema_tirolense

**Figure 27.**
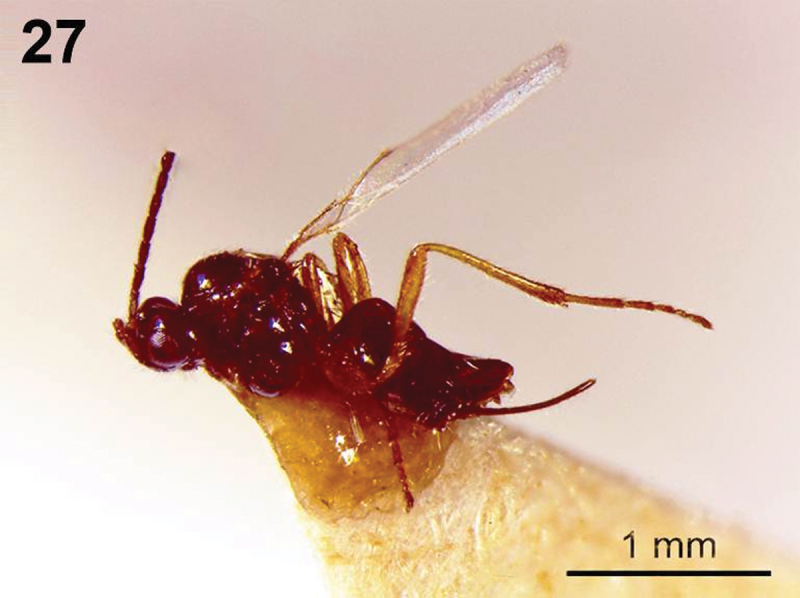
*Dinotrema tirolense* sp. n. (female). **27** Habitus, lateral view.

Figs 27 [Fig F8] –39

#### Type material.

Holotype: 1 female (ZSSM), “Italy, St. Peter/Ahrntal, Südtirol, 1950 m., Ja/26.08.1967, Haeselbarth”. Paratype: 1 female (ENV), same label as holotype but, “1800 m., Jh/26.08.1969, Haeselbarth”.

#### Diagnosis.

This new species resembles *Dinotrema sylvestre* Tobias but differs in having the mesoscutal pit present (absent in *Dinotrema sylvestre*), mandible 1.55–1.60 times as long as wide (as long as wide in *Dinotrema sylvestre*), first flagellar segment 4.25 times as long as wide (3.50 times in *Dinotrema sylvestre*), middle flagellar segments 3 times as long as their width (2.00 times in *Dinotrema sylvestre*) and hind femur 5.00 times as long as its maximum width (4.00 times in *Dinotrema sylvestre*).

#### Description.

Holotype, female, length of body 1.90–1.95 mm, of fore wing 3.00 mm.

*Head*. In dorsal view, 1.80 times as wide as its median length, 1.30–1.35 times as wide as mesoscutum, smooth, with rounded temples behind eye. Eye in lateral view 1.60–1.65 times as high as wide and 1.05–1.10 times as wide as temple. POL 2.85-2.90 times OD; OOL 2.75-2.80 times OD. Face 1.50-1.55 times as wide as high; inner margins of eyes subparallel. Clypeus 1.65 times as wide as high, slightly curved ventrally. Diameter of paraclypeal fovea less than half of distance between clypeus and eye. Mandible weakly widened towards apex, 1.55-1.60 times as long as its maximum width. Upper tooth distinctly shorter than middle tooth. Middle tooth the longest, widened basally and pointed apically, wider than upper and lower tooth. Lower tooth rounded apically and longer than upper tooth. Antenna thick, 23-segmented, as long as body. Scape 1.65-1.70 times as long as pedicel. First flagellar segment 4.25 times as long as its apical width, 1.25–1.30 times as long as second segment; second segment 3.00 times as long as its maximum width. Third to twentieth flagellar segments 3.00 times as long as their width; twenty-first segment 2.50 times as long as wide.

*Mesosoma*. In lateral view, 0.95 times as long as high. Mesoscutum as long as maximum width with three rows of two setae, two around notauli and one in middle part. Notauli mainly absent. Mesoscutal pit present, rounded. Prescutellar depression smooth, with small lateral carinae. Sternaulus (= precoxal suture) present, not reaching anterior and posterior parts of mesopleuron. Posterior mesopleural furrow smooth. Propodeum smooth, with incomplete median longitudinal carinae not crossing line of spiracles. Propodeal spiracles small.

*Legs*. Hind femur 5.00 times as long as wide. Hind tibia weakly widened to apex, 11.40 times as long as its maximum subapical width, 1.15–1.20 times as long as hind tarsus. First segment of hind tarsus 1.50 times as long as second segment.

*Wings*. Length of fore wing 2.30 times its maximum width. Vein r1 present. Radial cell reaching to apex of wing, 4.75 times as long as its maximum width. Nervulus distinctly postfurcal. Brachial cell closed, widened apically, 3.50 times as long as its maximum width. Hind wing 4.80–4.90 times as long as its maximum width.

*Metasoma*. Distinctly compressed. First tergite weakly widened towards apex, 1.60 times as long as its apical width, almost entirely smooth. Ovipositor 1.85-1.90 times as long as first tergite, shorter than metasoma, 1.15–1.20 times as long as hind femur.

*Colour*. Body and legs brown to dark brown. Wings hyaline. Pterostigma brown.

Male unknown.

#### Etymology.

The name is from geographical area “Tirol”, the type locality of species.

**Figures 28–33. F8:**
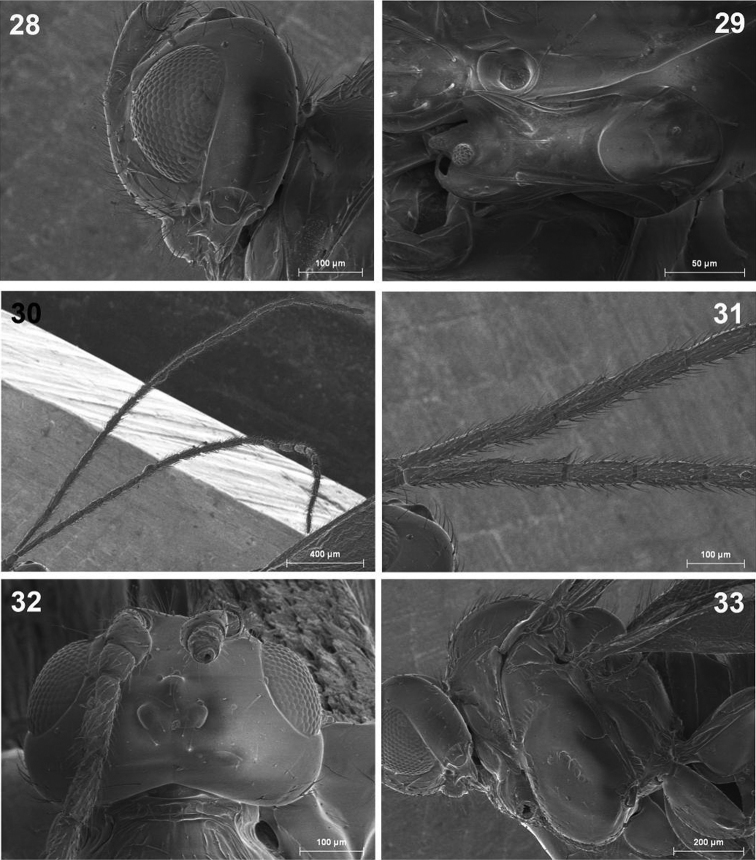
*Dinotrema tirolense* sp. n. (female). **28** Head, lateral view **29** Mandible **30** Antenna **31 **Basal segments of antenna **32** Head, dorsal view **33** Mesosoma.

**Figures 34–39.**
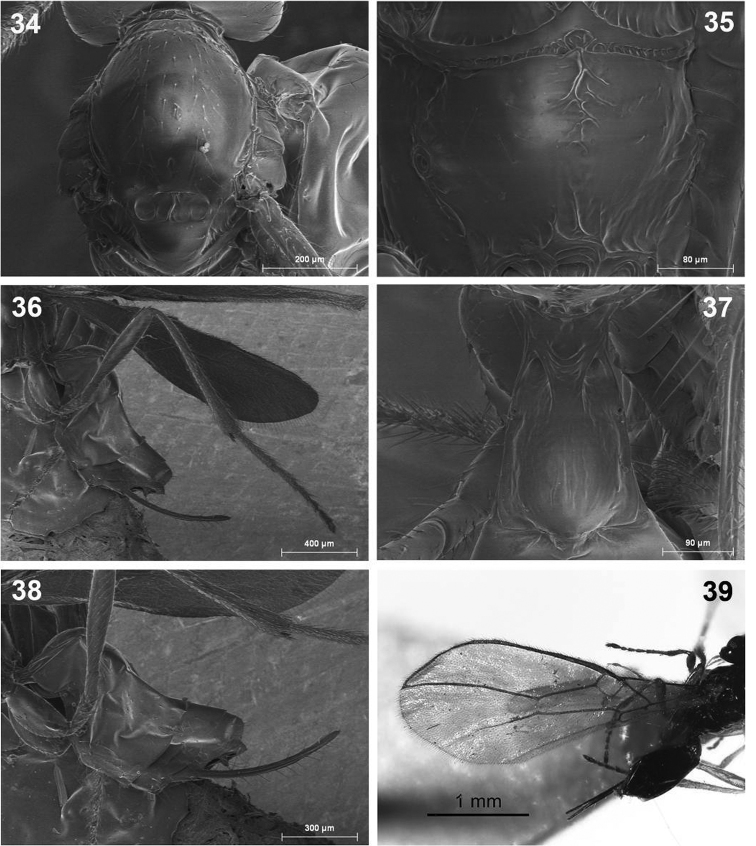
*Dinotrema tirolense* sp. n. (female). **34** Mesonotum **35** Propodeum **36** Hind leg **37 **First metasomal tergite **38** Metasoma and ovipositor **39** Fore wing.

### Dinotrema valvulatum

Munk & Peris-Felipo sp. n.

urn:lsid:zoobank.org:act:D3CEA3C7-32FB-411D-A479-AA9AEC125236

http://species-id.net/wiki/Dinotrema_valvulatum

Figs 40 –51

#### Type material.

Holotype: 1 female (NMA), “Denmark, E-Jutland, Højen Bæk, 5 km S of Vejle, 07.07.1984, Munk”. Paratype: 1 female (NMA), same label as holotype, both specimens were collected in a deciduous wood with *Alnus*, *Fraxinus* and *Fagus* on wet ground, 07.07.1984; 1 female (ZSSM), “Italy, St. Peter/Ahrntal, Südtirol, 1600 m., Ja/26.08.1967, Haeselbarth”.

#### Diagnosis.

This new species resembles *Dinotrema alysiae* sp. n.; their differences are given after the description of *Dinotrema alysiae*.

#### Description.

Holotype, female, Length of body 1.40–1.60 mm, of fore wing 2.20 mm.

*Head*. In dorsal view, 1.60 times as wide as its median length, 1.50 times as wide as mesoscutum, smooth, with rounded temples behind eye. Eye in lateral view 1.55 times as high as wide and 0.90–0.95 times as wide as temple. POL 2.75–2.80 times OD; OOL 3.40–3.45 times OD. Face 1.35 times as wide as high; inner margins of eyes subparallel. Clypeus 3.10 times as wide as high, slightly curved ventrally. Paraclypeal fovea large, its diameter more than half the distance between clypeus and eye. Mandible widened towards apex, 1.60 times as long as its maximum width. Upper tooth weakly shorter than middle tooth and wider than middle and lower tooth. Middle tooth the longest, widened basally and pointed apically. Lower tooth rounded apically and shorter than upper tooth. Antenna thick, 21-segmented, longer than body. Scape 2.00 times as long as pedicel. First flagellar segment 3.65–3.70 times as long as its apical width, 1.15 times as long as second segment; second segment 2.50 times as long as its maximum width. Third to eighteenth flagellar segments 2.20–2.30 times as long as their width; nineteenth segment 2.00 times as long as its maximum width.

*Mesosoma*. In lateral view, 1.10–1.15 times as long as high. Mesoscutum 1.10–1.15 times as long as maximum width with two rows of single setae. Notauli mainly absent. Mesoscutal pit present and elongated. Prescutellar depression smooth, without lateral carinae. Sternaulus (= precoxal suture) present, not reaching anterior and posterior parts of mesopleuron. Posterior mesopleural furrow smooth. Propodeum with median longitudinal carina running from anterior to posterior its margins, in anterior third with transverse angulated carina, with additional long subparallel carinae laterally to median one; from lateral carinae emerging short carinae not reaching propodeal edges. Propodeal spiracles relatively small.

*Legs*. Hind femur 4.50 times as long as wide. Hind tibia weakly widened to apex, 9.10–9.15 times as long as its maximum subapical width, as long as hind tarsus. First segment of hind tarsus 1.95–2.00 times as long as second segment.

*Wings*. Length of fore wing 2.50–2.60 times its maximum width. Vein r1 present. Radial cell reaching to apex of wing, 4.50 times as long as its maximum width. Nervulus distinctly postfurcal. Brachial cell closed, 3.00 times as long as its maximum width. Hind wing 8.0 times as long as its maximum width.

*Metasoma*. Distinctly compressed. First tergite weakly widened towards apex, 1.90 times as long as its apical width, almost sculptured with fine striae. Ovipositor 2.10 times as long as first tergite, as long as metasoma, 1.60–1.65 times as long as hind femur.

*Colour*. dark brown with a red tone, except propleuron, scapus, pedicellus and anterior half of metasoma infuscate reddish; clypeus and legs yellow (fifth tarsal segment infuscate).

Male unknown.

#### Etymology.

The name is due to the large size of the ovipositor valves.

**Figures 40–45.**
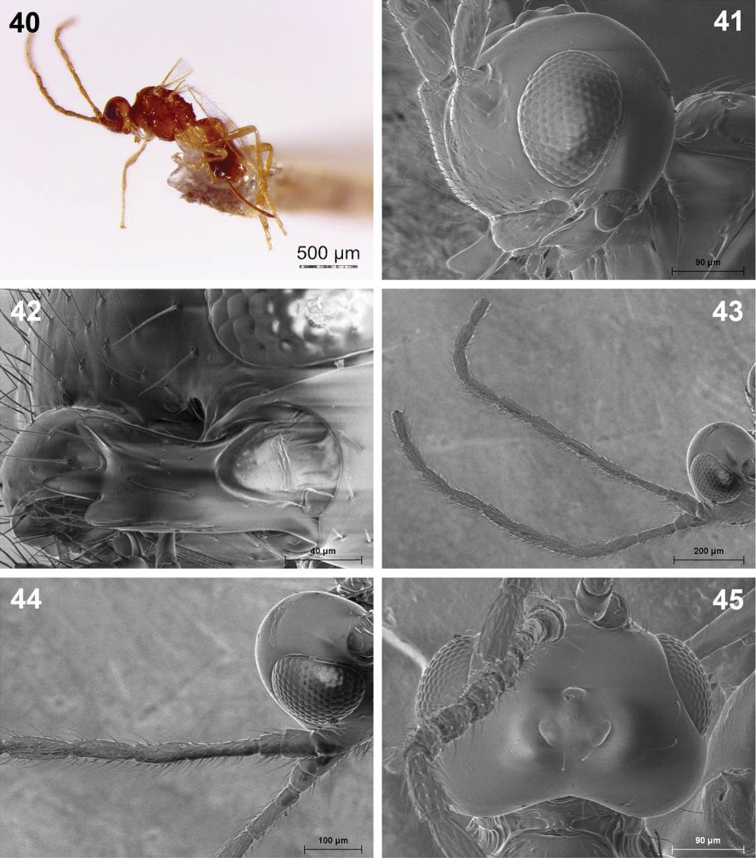
*Dinotrema valvulatum* sp. n. (female). **40** Habitus, lateral view **41** Head, lateral view **42** Mandible **43** Antenna **44** Basal segments of antenna **45** Head, dorsal view.

**Figures 46–51.**
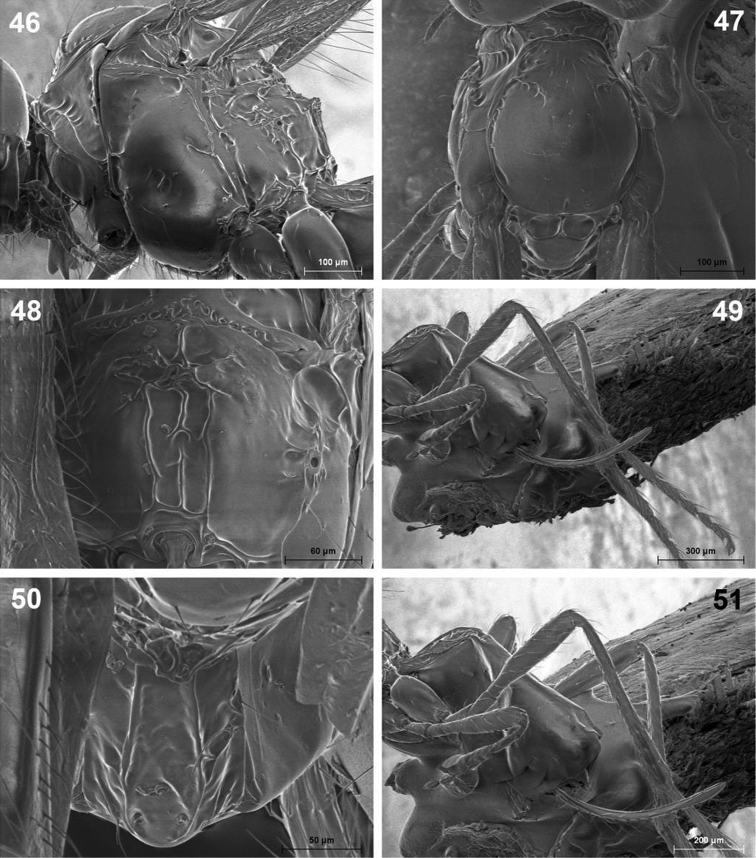
*Dinotrema valvulatum* sp. n. (female). **46** Mesosoma **47** Mesonotum **48** Propodeum **49** Hind leg **50** First metasomal tergite **51** Metasoma and ovipositor.

## Supplementary Material

XML Treatment for Dinotrema alysiae

XML Treatment for Dinotrema paramicum

XML Treatment for Dinotrema tirolense

XML Treatment for Dinotrema valvulatum
